# Non-destructive wood density assessment of Scots pine (*Pinus sylvestris* L.) using Resistograph and Pilodyn

**DOI:** 10.1371/journal.pone.0204518

**Published:** 2018-09-27

**Authors:** Irena Fundova, Tomas Funda, Harry X. Wu

**Affiliations:** 1 Umeå Plant Science Centre, Department of Forest Genetics and Plant Physiology, Swedish University of Agricultural Sciences, Umeå, Sweden; 2 Skogforsk (The Forestry Research Institute of Sweden), Sävar, Sweden; Technical University in Zvolen, SLOVAKIA

## Abstract

We tested two methods for non-destructive assessment of wood density of Scots pine standing trees: one based on penetration depth of a steel pin (Pilodyn) and the other on micro-drilling resistance (Resistograph). As a benchmark we used wood density data from x-ray analysis (SilviScan). We assessed in total 622 trees of 175 full-sib families growing in a single progeny test. Pilodyn was applied with bark (PIL) and without bark (PIL_B_). Raw Resistograph drilling profiles (RES) were adjusted (RES_TB_) in order to eliminate increasing trend caused by needle friction. Individual narrow-sense heritability of benchmark SilviScan density (DEN; 0.46) was most closely approached by that of adjusted RES_TB_ (0.43). Heritabilities were lower for unadjusted RES (0.35) as well as for PIL and PIL_B_ (both 0.32). Additive genetic correlations of the benchmark DEN with RES, RES_TB_, PIL and PIL_B_ were 0.89, 0.96, 0.59 and 0.71, respectively. Our results suggest that Resistograph is a more reliable tool than Pilodyn for wood density assessment of Scots pine; however, we highly recommend adjusting Resistograph drilling profiles prior to further analyses.

## Introduction

Scots pine (*Pinus sylvestris* L.) is a commercially significant forest tree species native to Eurasia. It is mainly used for production of sawn timber, pulp, and furniture [1]. In Sweden, Scots pine breeding started in 1950s with phenotypic selection of superior trees, followed by establishment of seed orchards. Emphasis was mainly put on growth, vitality, branching habit and stem straightness, whereas wood quality traits such as density or stiffness were not considered [2]. Unfortunately, negative relationship between growth and wood quality traits has been reported in a number of conifer tree species including Scots pine [3, 4], radiata pine [5], maritime pine [6], black spruce [7] or Douglas-fir [8] and it is therefore of vital importance to include wood quality traits in breeding programs too. This task brings about a need to find reliable tools for non-destructive wood quality assessment that would be capable of a rapid evaluation of a large number of standing trees.

Wood density is considered to be the best single predictor of wood quality. It is well correlated with other wood quality traits such as strength or stiffness [9] and it significantly affects wood suitability for different end uses. For instance, higher wood density is more appropriate for constructional lumber, is associated with greater pulp yield [6] and increases wood machinability, particularly boring, shaping and mortising [10].

Wood density is defined as the mass of wood per unit volume at a given moisture content. It can be accurately determined by either traditional volumetric method [11] or x-ray densitometry [12]. X-ray densitometry combined with x-ray diffraction and image analysis is incorporated in SilviScan technology, which enables efficient measuring of numerous wood and fiber properties such as wood density, stiffness, microfibril angle or fiber dimensions [13]. However, both the volumetric and x-ray methods are enormously time, cost and labor intensive and, in addition, require extracted increment core samples; thus, they cannot be classified as truly non-destructive.

Several, to a lesser or greater extent, non-destructive methods have been developed for rapid field wood density assessment. They include quantification of 1- torque of a borer (torsiometers), 2- withdrawal resistance of a nail, 3- penetration depth of a pin (penetrometers) or 4- micro-drilling resistance (resistometers) [14]. Penetrometers and micro-drilling resistometers use a thin steel probe that penetrates into wood, leaving just a slight hole. They were originally developed for testing the quality of wooden structures, but have proven to be suitable for wood density assessment of standing trees as well. The Pilodyn penetrometer measures penetration depth of a spring-loaded blunt pin (Ø 2 mm) that is shot into the wood with an exact force. Its records give strong genetic correlations with wood density in some conifer species [15–18]; however, the penetration depth is limited to a few centimeters of the outer wood (2–3 cm) and, hence, no information is provided about the rest of a tree’s profile. Moreover, it is also worthwhile to consider removing bark prior the measurement [19], as the bark may constitute a substantial portion of the assessed profile and its uneven thickness and lower density might affect the estimates. On the other hand, the Resistograph micro-drill accounts for the whole stem’s profile at a given height, as it measures drilling resistance, i.e., energy needed for a drill needle (Ø 3 mm) to penetrate wood, from bark to bark at a constant speed [20]. A resistogram, a visualized profile of drilling resistance plotted against penetration depth, reveals density variation inside a stem induced by alternating earlywood and latewood as well as by the presence of knots, cavities, or wood in various stages of physical deterioration. Resistograms closely resemble x-ray density profiles; they have similar sensitivity although their local resolution is a little lower. Their drawback is that they often exhibit an increasing trend caused by accumulated needle friction [21]; therefore, they should be detrended to eliminate bias in wood density estimates [12]. The necessity of such post-measurement adjustment is probably the main reason why the Resistograph has not been widely used for wood density assessment in forest genetics field tests [14]. However, strong genetic correlations between wood density and mean Resistograph density have been reported by several studies [8, 12, 22], implying that the Resistograph could be a suitable tool for wood density assessment also in Scots pine.

The aim of this study is to (1) develop an algorithm suitable for Scots pine to determine mean density from a Resistograph’s drilling profile; (2) evaluate the reliability of Resistograph and Pilodyn measurements for wood density assessment in Scots pine using SilviScan data as a benchmark; (3) estimate inheritance of Resistograph and Pilodyn wood density measurements; and (4) calculate their phenotypic and genetic correlations with growth and wood quality traits.

## Materials and methods

### Test material

The study was conducted in a Scots pine (*Pinus sylvestris* L.) full-sib progeny test #S23F 711261 “Grundtjärn” (lat. 63.5556° N, long. 17.4139° E, alt. 320 m, area 3.5 ha) located in central Sweden. The test was established for research purposes by The Forestry Research Institute of Sweden (Skogforsk) in 1971 on silty moraine using completely randomized single tree plot design. It comprised of 7,240 trees representing 179 full-sib families that were generated with a partial diallel mating design using 45 parents. Geographic origin of the parents (plus trees) and mating design for this progeny test was described in [3]. The test site was divided into 181 post-blocks to ease orientation and remove environmental effects during data analyses, and each post-block consisted of 40 trees (4 columns by 10 rows with spacing of 2.2 m in both directions). In total 622 trees representing 175 families of 44 parents were included in this study.

Skogforsk, an organization that has the authority to grant permissions to access and maintain this progeny test as well as collect, analyze and publish any data generated therein, approved this study; no other permissions were required. No protected or endangered species were involved.

### *In situ* wood density measurement

The micro-drill Resistograph IML-RESI PD300 (Instrumenta Mechanic Labor, Germany) and penetrometer Pilodyn 6J Forest (PROCEQ, Switzerland) were used for estimating wood density of standing trees. The Resistograph was used for drilling trees bark to bark in two mutually perpendicular directions (from southeast and from southwest) at height of ca 1.2 m and special attention was paid to avoiding drilling through knots or visible stem damages. Each profile was checked immediately after drilling on the tool’s screen and the measurement was repeated when necessary. Pilodyn was applied on each stem with bark and without bark in the southwest direction at ca 1.3 m above ground. Both the Resistograph and Pilodyn (with bark) were applied on fissures between bark scales to ensure that the tools’ tips were stable during measurements and to avoid high proportion of bark in the records. The reciprocal of Pilodyn’s penetration depth and an average value of adjusted Resistograph’s records were used as indirect wood density estimates.

### Processing of resistograms

**Fig 1:**
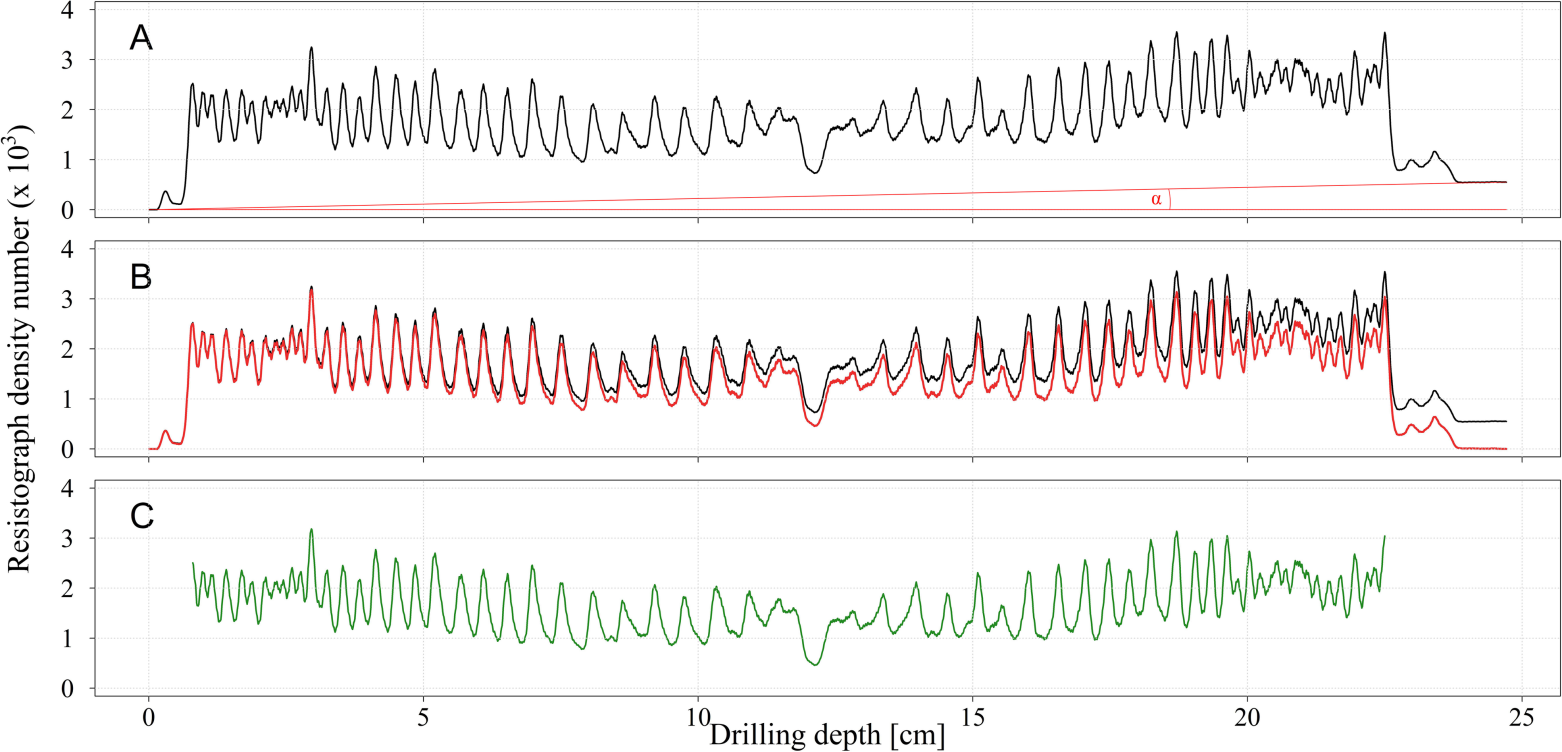
Unadjusted (A), detrended (B), and detrended & debarked (C) Resistograph profiles.

**Fig 2:**
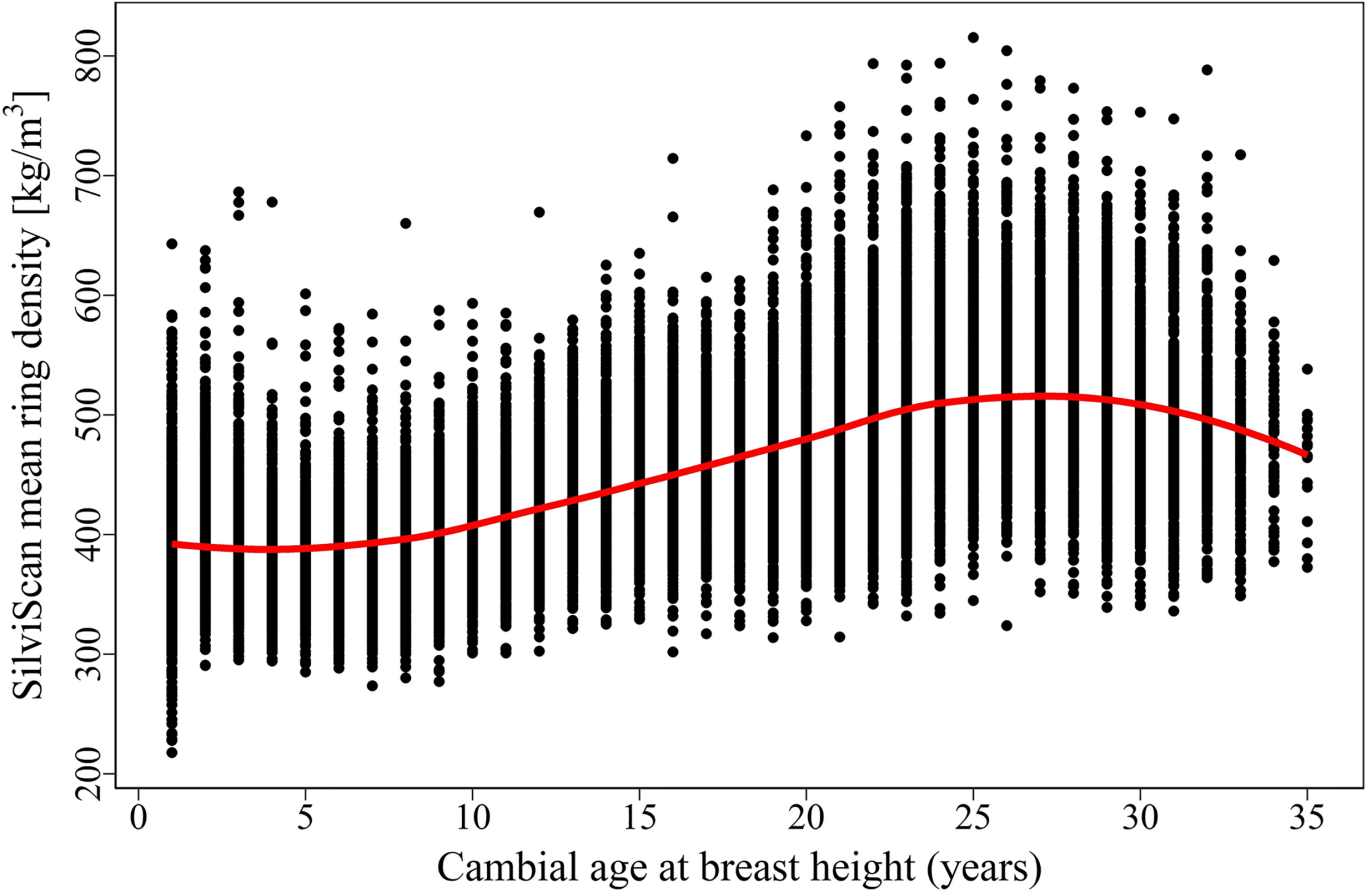
SilviScan mean ring density plotted against cambial age.

The Resistograph IML-RESI PD300 is capable of measuring trees up to 30.2 cm in diameter with a drilling resolution of 100 points per centimeter. Each radial measurement generates a resistogram that includes lower-density bark on both sides and a pith in the center (Fig 1). The overall shape of a resistogram also reveals differences in juvenile and mature wood densities, resulting in a bowl-like shape in the center passing into a plateau or slightly decreasing at the edges, which is typical for conifers [21]; a smooth curve fitted through a scatter plot of SilviScan mean ring density and cambial age makes the natural shape of wood density variation more clearly visible (Fig 2). Aside from the natural shape, most of the Resistograph profiles exhibited an increasing trend caused by accumulated needle friction (Fig 1A).

In order to eliminate bias in wood density estimates, each profile with this issue was detrended, assuming a linear trend. A slope of increase was calculated for each profile separately as

$$\tan\;\alpha =\frac{{DN}_{R_{tail}}-{DN}_{R_{head}}}{L_{max}}$$ (1)

where ${DN}_{R_{tail}}$ and ${DN}_{R_{head}}$ are Resistograph density numbers at the tail and head of a raw profile, respectively, and *L*_*max*_ is the total length of the profile. Then every density number point (100 cm^-1^) along the whole profile was adjusted as

$${DN}_{T_{i}}={DN}_{R_{i}}-(L_{i}\times \tan\alpha )$$ (2)

where ${DN}_{R_{i}}$ and ${DN}_{T_{i}}$ are Resistograph density numbers of the raw and detrended profiles at point *i*, respectively, and *L*_*i*_ is the length of the profile from the beginning till position *i* (Fig 1B). When a Resistograph profile was incomplete, whether due to the stem diameter being too large or due to an abrupt profile ending, the density number was detrended based on the overall-site average slope. Bark, together with the first unfinished growth ring, was removed from each detrended profile in order to obtain a more accurate wood density estimate (Fig 1C). The average density number (Resistograph wood density, RES) was calculated as the arithmetic mean of all density numbers *DN*_*i*_ along a profile. Resistograph wood density was calculated for each raw, detrended, and detrended & debarked profile and for each of the two drilling directions separately (the southeastern and southwestern sides being marked as A and B, respectively) and combined.

### SilviScan data

Bark-to-bark increment cores with diameter of 10 mm were taken at 1.3 m from southeastern side of each stem in 2011. Prior to the SilviScan (CSIRO, Australia) analysis, pith-to-bark radial strips (2 mm thick and 7 mm wide) were sawn from the cores, soaked in acetone to remove extractives and air-dried under laboratory conditions (23°C and 43% relative humidity). The following traits, obtained from SilviScan analysis, were used in this study: mean wood density (DEN); density of earlywood (EWD), transition wood (TWD) and latewood (LWD); proportion of earlywood (EWP), transition-wood (TWP) and latewood (LWP); microfibril angle (MFA); static modulus of elasticity (MOEs); and fiber wall thickness (FWT), fiber coarseness (FCS) and fiber width in radial (FRW) and tangential (FTW) direction. DEN served as a benchmark for evaluation of the Resistograph and Pilodyn for indirect wood density measurement. Earlywood, transition wood and latewood of each growth ring were defined as sections with densities ranging from 0-20%, 20-80% and 80-100% of the total density range within the annual ring (minimum to maximum), respectively. Since mean values weighted by the ring areas were shown to more accurately represent average wood properties [23], area-weighted values (*AWV*) for fiber and wood quality traits were calculated as:

$$AWV=\frac{\sum \left(\alpha_{i}d_{i}\right)}{\sum \alpha_{i}}$$ (3)

where *d*_*i*_ is a value for annual ring *i* with an area *α*_*i*_ [24].

### Growth traits

Height (HGT) and diameter at breast height (DBH) were measured in summer 2011 and stem volume (VOL) was calculated according to [25] as a function of height and diameter.

### Statistical analysis

The response variables were fitted into the following linear mixed model using statistical package ASReml 4 [26]:

$$y_{ijkl}=\mu +B_{i}+G_{j}+G_{k}+S_{jk}+e_{ijkl}$$ (4)

where *y*_*ijkl*_ is the *l*th observation for an offspring of *j*th and *k*th parent growing in *i*th block, *μ* is the overall mean of a given variable, *B*_*i*_ is random effect of *i*th block, *G*_*j*_ and *G*_*k*_ are random general combining ability effects of the *j*th and *k*th parent, respectively, *S*_*jk*_ is random specific combining ability effect for the cross between parents *j* and *k*, and *e*_*ijkl*_ is random error term.

Individual-tree narrow-sense heritabilities ($h_{i}^{2}$) for each variable were estimated using variance components from the univariate analysis as

$$h_{i}^{2}=\frac{\sigma_{A}^{2}}{\sigma_{P}^{2}}=\frac{{4\sigma }_{G}^{2}}{{2\sigma }_{G}^{2}+\sigma_{S}^{2}+\sigma_{e}^{2}}$$ (5)

where $\sigma_{A}^{2},\;\sigma_{P}^{2},\;\sigma_{G}^{2},\;\sigma_{S}^{2}$, and $\sigma_{e}^{2}$ are variances for additive genetic, phenotypic, general combining ability, specific combining ability, and residual components, respectively. Standard errors were obtained using Taylor series expansion [26]. Phenotypic and genetic correlations (*r*_*xy*_) were calculated as

$$r_{xy}=\frac{\sigma_{xy}}{\sqrt{\sigma_{x}^{2}\times \sigma_{y}^{2}}}$$ (6)

where $\sigma_{x}^{2}$ and $\sigma_{y}^{2}$ are phenotypic or additive genetic variance components for traits *x* and *y*, respectively, and *σ*_*xy*_ is phenotypic or additive genetic covariance component between traits *x* and *y* estimated by fitting a bivariate mixed model (Eq 4) [26]. Significance of the correlation coefficients was examined using *t*-test

$$t=r_{xy}\sqrt{\frac{n-2}{1-r_{xy}^{2}}}$$ (7)

where *t* is a *t*-value with *n*-2 degrees of freedom, *n* is the number of pairs and *r*_*xy*_ is the Pearson product-moment correlation. Furthermore, hierarchical cluster analysis based on dissimilarity matrix of additive genetic correlations between traits was performed using hclust function in R program [27] in order to construct an illustrative dendrogram. Genetic gain ($G_{A_{x}}$) for direct selection was calculated as

$$G_{A_{x}}=ih_{x}^{2}{CV}_{x}$$ (8)

where *i* is selection intensity (1% = 2.665), $h_{x}^{2}$ is individual-tree narrow-sense heritability for trait *x*, and *CV*_*x*_ is coefficient of variation for trait *x* calculated as phenotypic standard deviation divided by the mean. Correlated response (*CR*_*y*_) to selection for a target trait *y* was calculated as

$${CR}_{y}=ih_{x}h_{y}r_{xy}{CV}_{y}$$ (9)

where *h*_*x*_ and *h*_*y*_ are square roots of narrow sense heritabilities for selection trait *x* and target trait *y*, respectively, *r*_*xy*_ is the genetic correlation between trait *x* and *y*, and *CV*_*y*_ is the coefficient of variation for target trait *y*.

## Results

### Variation in the studied traits

Descriptive statistics for wood density estimated by Resistograph, Pilodyn and SilviScan as well as for other wood, fiber and growth traits are summarized in Table 1. SilviScan area-weighted wood density (DEN), used as a benchmark trait in our evaluation study, varied between 365 and 566 kg.m^-3^, with mean value of 448 kg.m^-3^. On average, wood contained a substantially higher proportion of earlywood (55%) than latewood (15%). The Resistograph unadjusted wood density (RES) ranged from 1242 to 2403 density units from side A, and from 1427 to 2492 density units from side B. Measurements from side A showed a slightly higher variation (10.7%) compared to side B (9.7%). Linear detrending of resistograms lowered wood density estimates, while debarking increased them since the lower-density bark was excluded (RES_T_ = 1644 < RES_TB_ = 1848 < RES = 1857). Pilodyn penetration depth measured with bark (PIL) ranged from 15 to 31 mm with an average of 21.7 mm, and it decreased after bark removal (PIL_B_) to only between 11 and 22 mm with an average of 16.4 mm. Variation of Pilodyn penetration depth was slightly higher after bark removal (11.4%). VOL exhibited the highest variation among the measured traits (34.5%), followed by MFA (23.8%).

**Table 1:** List of variables and descriptive statistics–minimum, maximum, mean, standard deviation (SD), coefficient of variation (CV) and individual narrow-sense heritabilities ($h_{i}^{2}$) with standard errors in parentheses.

Trait	Units	Description	*Min*	*Max*	*Mean*	*SD*	*CV*	$h_{i}^{2}$
*Wood density traits*								
RES_A_	-	Resistograph unadjusted density from SE	1242.4	2403.1	1836.6	197.1	10.7	0.29 (0.09)
RES_B_	-	Resistograph unadjusted density from SW	1427.3	2492.3	1878.0	182.5	9.7	0.31 (0.09)
RES	-	Resistograph mean unadjusted density	1354.4	2365.9	1857.4	140.3	8.5	0.35 (0.10)
RES_T-A_	-	Resistograph detrended density from SE	1135.4	2122.4	1632.5	155.7	9.5	0.36 (0.10)
RES_T-B_	-	Resistograph detrended density from SW	1270.9	2096.2	1655.0	142.9	8.6	0.42 (0.10)
RES_T_	-	Resistograph mean detrended density	1209.1	2041.9	1643.9	140.3	8.5	0.44 (0.11)
RES_TB-A_	-	Resistograph detrended and debarked density from SE	1246.8	2481.0	1838.9	183.4	10.0	0.36 (0.10)
RES_TB-B_	-	Resistograph detrended and debarked density from SW	1403.5	2388.1	1856.5	169.4	9.1	0.43 (0.11)
RES_TB_	-	Resistograph mean detrended and debarked density	1325.9	2351.6	1847.8	166.9	9.0	0.43 (0.11)
PIL	mm	Depth of Pilodyn's pin penetration with bark	15.0	31.0	21.7	2.3	10.8	0.32 (0.09)
PIL_B_	mm	Depth of Pilodyn's pin penetration without bark	11.0	22.0	16.4	1.9	11.4	0.32 (0.09)
DEN	kg∙m^-3^	SilviScan mean density	364.8	566.1	447.9	33.1	7.4	0.46 (0.10)
EWD	kg∙m^-3^	SilviScan density of earlywood	280.0	403.4	332.9	23.1	6.9	0.40 (0.10)
TWD	kg∙m^-3^	SilviScan density of transition wood	411.4	624.0	507.2	34.3	6.8	0.50 (0.10)
LWD	kg∙m^-3^	SilviScan density of latewood	595.3	894.6	751.4	51.8	6.9	0.51 (0.11)
*Other wood and fiber traits*								
EWP	%	Proportion of earlywood	40.3	67.2	55.3	4.3	7.8	0.16 (0.06)
TWP	%	Proportion of transition wood	18.2	44.3	29.5	4.2	14.2	0.12 (0.05)
LWP	%	Proportion of latewood	9.2	22.4	15.3	2.0	12.9	0.27 (0.08)
MFA	°	Microfibril angle	8.5	33.2	17.3	4.1	23.8	0.30 (0.08)
MOE_s_	GPa	Modulus of elasticity measured by SilviScan	5.0	16.0	10.2	1.9	18.5	0.39 (0.09)
FWT	μm	Fibre wall thickness	1.9	3.1	2.5	0.2	7.9	0.53 (0.11)
FCS	μg∙m^-1^	Fibre coarseness	297.2	505.6	406.4	33.3	8.2	0.57 (0.11)
FRW	μm	Fibre width in radial direction	25.8	32.0	28.7	1.1	3.7	0.46 (0.10)
FTW	μm	Fibre width in tangential direction	28.7	38.4	33.5	1.4	4.2	0.55 (0.11)
*Growth traits*								
DBH	cm	Diameter at breast height at age 40	8.2	29.4	19.7	3.2	16.3	0.22 (0.07)
HGT	m	Height at age 40	12.2	19.6	16.3	1.3	8.1	0.35 (0.09)
VOL	dm^3^	Stem volume at age 40	35.7	520.0	251.4	86.8	34.5	0.22 (0.07)

**Note:** Variables DEN, EWD, TWD, LWD, MFA, MOE_s_, FWT, FCS, FRW, FTW are area-weighted SilviScan measurements.

### Heritability

Individual-tree narrow-sense heritability of Resistograph wood density substantially increased after detrending of raw density profiles (from 0.35 to 0.44), reaching a similar value as that of the benchmark variable DEN (0.46) obtained from SilviScan (Table 1). Removing bark however did not bring any further improvement. Pilodyn density heritability PIL (0.32) was close to that attained by the Resistograph using raw data (0.35), but since cutting off bark prior to the measurements did not bring any improvement to the estimate either (PIL_B_ remained at 0.32), the Resistograph proved to be a superior tool in this regard. Fiber traits exhibited highest heritabilities, particularly FCS (0.57), FTW (0.55) and FWT (0.53), followed by LWD (0.51). Compared to wood and fiber traits, growth traits had lower heritabilities (0.22–0.35).

### Phenotypic and genetic correlations of indirect wood density measurements with SilviScan wood density as a benchmark

**Fig 3:**
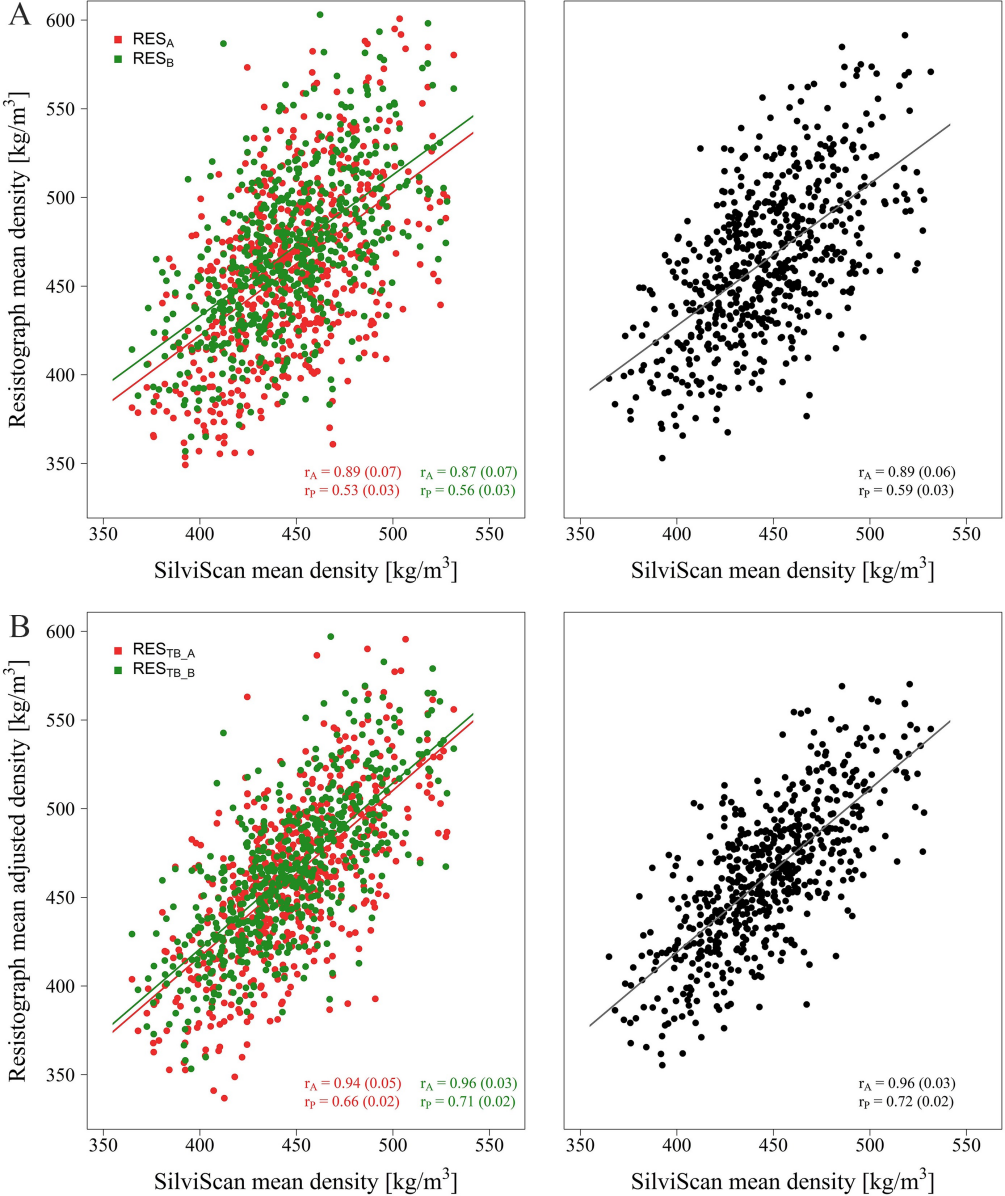
Relationship between SilviScan area-weighted mean density (DEN) and (A) unadjusted Resistograph mean density (RES_A_ and RES_B_ on the left, RES on the right) and (B) adjusted Resistograph mean density (RES_TB_A_ and RES_TB_B_ on the left, RES_TB_ on the right).

**Fig 4:**
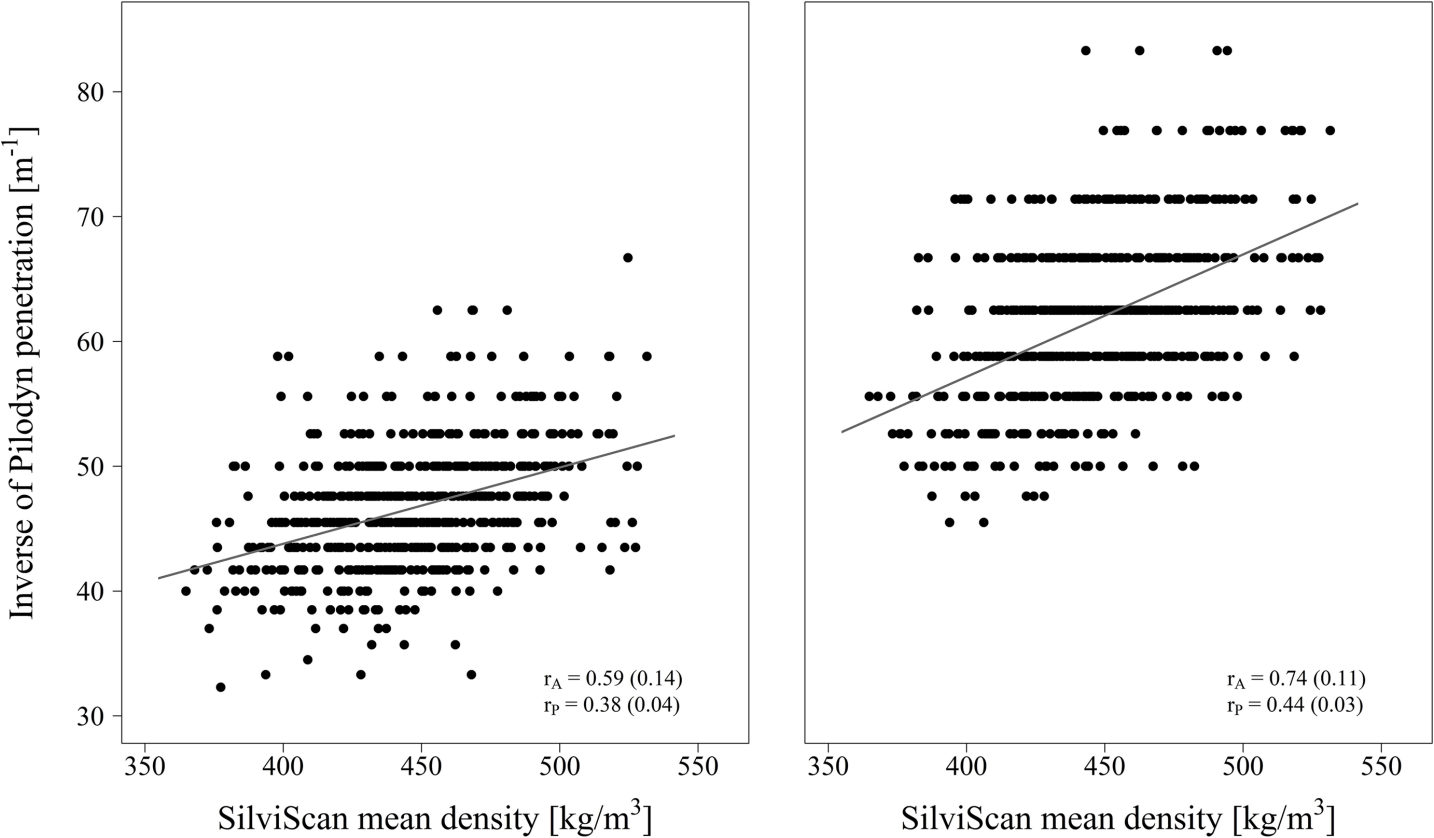
Relationship between SilviScan area-weighted mean density (DEN) and inverse value of Pilodyn penetration depth with bark (left) and without bark (right).

Phenotypic correlations of unadjusted density RES with the benchmark DEN were moderate (0.53, 0.56 and 0.59 for side A, B and mean of the two sides, respectively) (Fig 3A, Table 2) but they considerably improved following detrending (RES_T_; 0.63, 0.68 and 0.69, respectively) and even slightly more after subsequent debarking (RES_TB_; 0.66, 0.71, and 0.72, respectively; Fig 3B). Compared to phenotypic correlations, genetic correlations showed only minor differences between the two sides and their mean values. Genetic correlations of the benchmark DEN with mean RES, RES_T_ and RES_TB_ increased from 0.89 through 0.93 up to 0.96, respectively. Phenotypic and genetic correlations between DEN and PIL_B_ (0.44 and 0.74, respectively) were higher than between DEN and PIL (only 0.38 and 0.59, respectively) (Fig 4, Table 2).

**Table 2:** Genetic and phenotypic correlations of SilviScan area-weighted mean density (DEN) with different Resistograph and Pilodyn wood density estimates (standard errors in parentheses).

	Correlations with DEN	
	genetic	phenotypic
RES	0.89 (0.06)	0.59 (0.03)
RES_A_	0.89 (0.07)	0.53 (0.03)
RES_B_	0.87 (0.07)	0.56 (0.03)
RES_T_	0.93 (0.04)	0.69 (0.02)
RES_T-A_	0.90 (0.06)	0.63 (0.03)
RES_T-B_	0.93 (0.04)	0.68 (0.02)
RES_TB_	0.96 (0.03)	0.72 (0.02)
RES_TB-A_	0.94 (0.05)	0.66 (0.02)
RES_TB-B_	0.96 (0.03)	0.71 (0.02)
PIL	0.59 (0.14)	0.38 (0.04)
PIL_B_	0.74 (0.11)	0.44 (0.03)

All correlations are statistically significant at α = 0.01.

### Phenotypic and genetic correlations of indirect wood density measurements with other wood, fiber and growth traits

**Fig 5:**
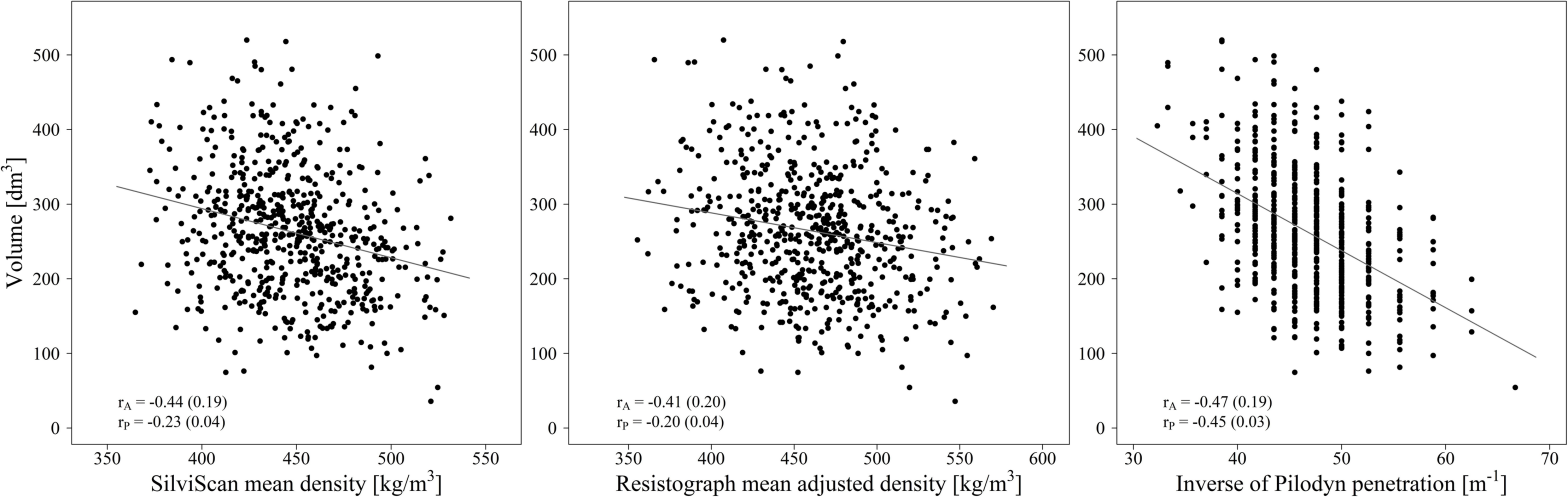
Relationships of stem volume (VOL) with SilviScan (DEN), Resistograph (RES_TB_) and Pilodyn (PIL) wood density.

**Fig 6:**
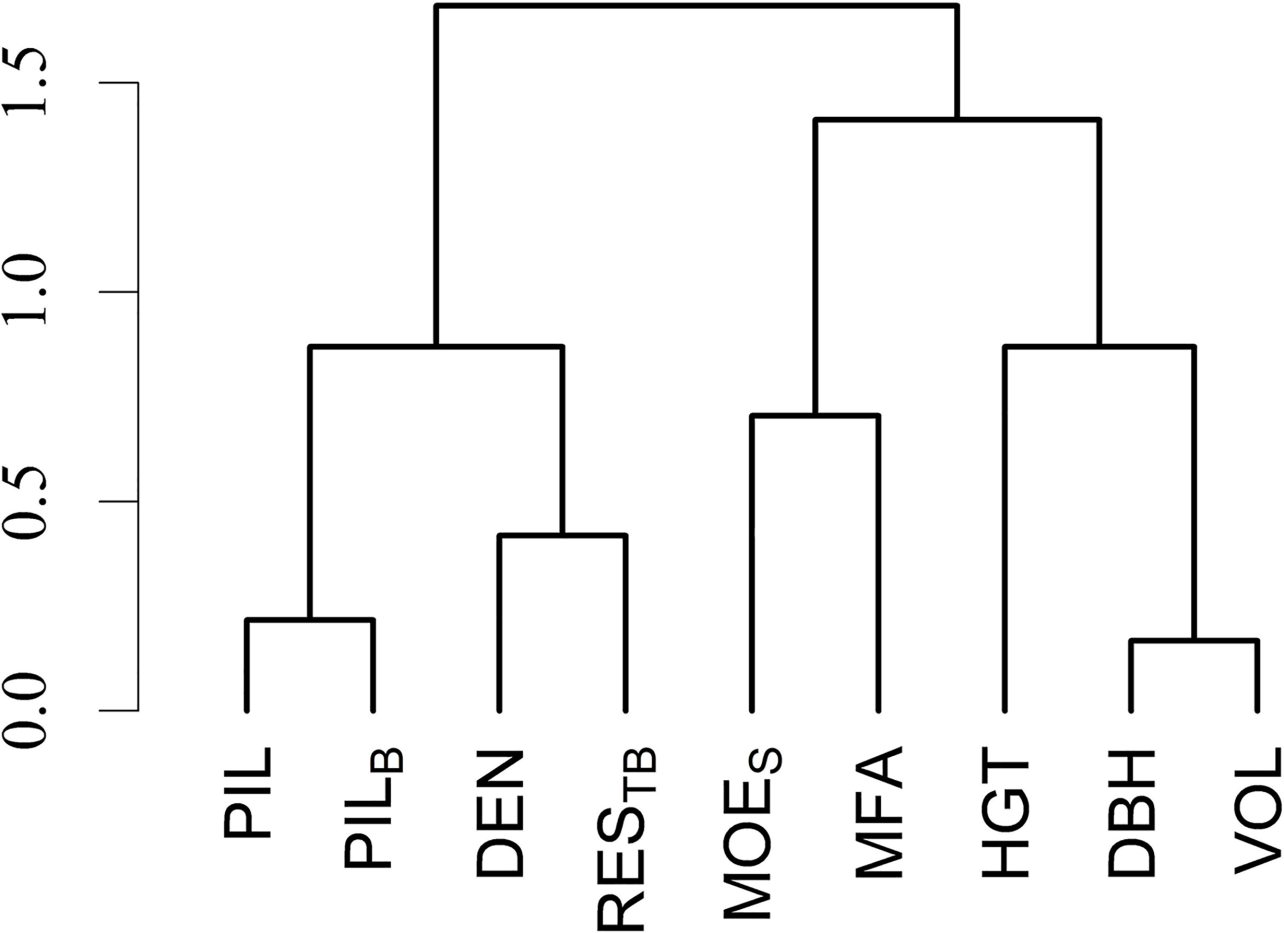
Dendrogram of genetic relationships among growth and wood quality traits.

Phenotypic and genetic correlations of the benchmark DEN and the indirect wood density variables (RES, RES_T_, RES_TB_, PIL and PIL_B_) with other wood, fiber and growth traits are summarized in Table 3. In most cases, the adjusted Resistograph wood density gave stronger correlations associated with lower standard errors compared to unadjusted measurements ($r_{RES}<r_{{RES}_{T}}<r_{{RES}_{TB}}$). Strong positive genetic correlations between Resistograph wood density and the three wood density components (EWD, TWD and LWD) reflected those with benchmark DEN, although they were a little lower. Genetic correlations of the three components with Pilodyn measurements were moderate, but PIL_B_ had a stronger relationship than PIL. Phenotypic correlations of the components were strong with DEN, moderate with Resistograph measurements and weak with Pilodyn measurements. Correlations with FWT followed a similar pattern. The indirect wood density estimates, including DEN, had strong to moderate positive correlations with LWP but their correlations with EWP and TWP were negative, whereas genetic correlations were in many cases non-significant and phenotypic correlations were associated with high standard errors. DEN showed moderate negative genetic and weak negative phenotypic correlations with MFA. Both genetic and phenotypic correlations of MFA with Resistograph and Pilodyn measurements were very weak. Moderate genetic and weak phenotypic correlations between MOE_s_ and Resistograph and Pilodyn measurements were lower than those with the benchmark DEN. Growth traits generally correlated negatively with DEN as well as with the indirect wood density estimates (Fig 5). Genetic correlations with DBH and VOL were moderate, while correlations with HGT were weak. Relationships among growth and wood quality traits based on additive genetic correlations are visualized in Fig 6.

**Table 3:** Genetic and phenotypic correlations of indirect wood density estimates (SilviScan, Resistograph and Pilodyn) with growth, fiber and other wood quality traits (standard errors in parentheses).

	Genetic correlations						Phenotypic correlations					
	DEN	RES	RES_T_	RES_TB_	PIL	PIL_B_	DEN	RES	RES_T_	RES_TB_	PIL	PIL_B_
	*Wood traits*											
EWD	0.87 ** (0.05)	0.78 ** (0.10)	0.83 ** (0.07)	0.85 ** (0.07)	0.48 ** (0.17)	0.57 ** (0.15)	0.90 ** (0.01)	0.47 ** (0.03)	0.59 ** (0.03)	0.63 ** (0.03)	0.35 ** (0.04)	0.37 ** (0.04)
TWD	0.95 ** (0.02)	0.82 ** (0.08)	0.84 ** (0.07)	0.86 ** (0.06)	0.43 ** (0.17)	0.60 ** (0.14)	0.90 ** (0.01)	0.54 ** (0.03)	0.65 ** (0.03)	0.67 ** (0.02)	0.34 ** (0.04)	0.38 ** (0.04)
LWD	0.92 ** (0.03)	0.72 ** (0.11)	0.77 ** (0.09)	0.80 ** (0.08)	0.41 ** (0.18)	0.60 ** (0.14)	0.77 ** (0.02)	0.41 ** (0.04)	0.54 ** (0.03)	0.57 ** (0.03)	0.34 ** (0.04)	0.36 ** (0.04)
EWP	-0.17 ** (0.22)	-0.23 ** (0.24)	-0.25 ** (0.23)	-0.27 ** (0.23)	-0.41 ** (0.23)	-0.41 ** (0.22)	-0.22 ** (0.04)	-0.22 ** (0.04)	-0.19 ** (0.04)	-0.17 (0.04)	-0.06 ^ns^ (0.04)	-0.15 * (0.04)
TWP	-0.29 ** (0.24)	-0.20 ** (0.27)	-0.15 * (0.26)	-0.15 * (0.26)	0.28 ** (0.27)	0.22 ** (0.27)	-0.03 ^ns^ (0.04)	0.07 ^ns^ (0.04)	0.01 ^ns^ (0.04)	-0.03 ^ns^ (0.04)	-0.05 ^ns^ (0.04)	0.04 ^ns^ (0.04)
LWP	0.76 ** (0.10)	0.72 ** (0.12)	0.72 ** (0.12)	0.76 ** (0.11)	0.45 ** (0.19)	0.60 ** (0.16)	0.54 ** (0.03)	0.41 ** (0.03)	0.41 ** (0.03)	0.44 ** (0.03)	0.23 ** (0.04)	0.22 ** (0.04)
MFA	-0.52 ** (0.16)	-0.14 * (0.22)	-0.21 ** (0.20)	-0.21 ** (0.20)	-0.19 ** (0.21)	-0.08 * (0.21)	-0.24 ** (0.04)	0.03 ^ns^ (0.04)	-0.09 * (0.04)	-0.13 * (0.04)	-0.17 ** (0.04)	-0.10 * (0.04)
MOE_s_	0.84 ** (0.07)	0.52 ** (0.16)	0.59 ** (0.14)	0.61 ** (0.14)	0.44 ** (0.18)	0.43 ** (0.18)	0.60 ** (0.03)	0.21 ** (0.04)	0.36 ** (0.04)	0.41 ** (0.04)	0.33 ** (0.04)	0.26 ** (0.04)
	*Fiber traits*											
FWT	0.90 ** (0.04)	0.78 ** (0.09)	0.80 ** (0.08)	0.84 ** (0.07)	0.43 ** (0.17)	0.60 ** (0.14)	0.92 ** (0.01)	0.55 ** (0.03)	0.63 ** (0.03)	0.64 ** (0.03)	0.31 ** (0.04)	0.36 ** (0.04)
FCS	0.59 ** (0.12)	0.47 ** (0.16)	0.49 ** (0.16)	0.51 ** (0.15)	0.18 ** (0.20)	0.30 ** (0.19)	0.65 ** (0.02)	0.41 ** (0.04)	0.43 ** (0.04)	0.42 ** (0.04)	0.16 ** (0.04)	0.20 ** (0.04)
FRW	-0.30 ** (0.18)	-0.27 ** (0.20)	-0.38 ** (0.18)	-0.40 ** (0.17)	-0.46 ** (0.18)	-0.42 ** (0.18)	-0.12 * (0.04)	-0.07 ^ns^ (0.04)	-0.10 * (0.04)	-0.12 *(0.04)	-0.12 * (0.04)	-0.15 * (0.04)
FTW	-0.17 ** (0.19)	-0.27 ** (0.20)	-0.17 ** (0.20)	-0.16 ** (0.20)	-0.22 ** (0.20)	-0.15 * (0.20)	-0.35 ** (0.04)	-0.20 ** (0.04)	-0.31 ** (0.04)	-0.35 ** (0.04)	-0.28 ** (0.04)	-0.24 ** (0.04)
	*Growth traits*											
DBH	-0.47 ** (0.18)	-0.22 ** (0.24)	-0.37 ** (0.21)	-0.45 ** (0.19)	-0.54 ** (0.18)	-0.50 ** (0.19)	-0.27 ** (0.04)	0.18 ** (0.04)	-0.11 * (0.04)	-0.25 ** (0.04)	-0.50 ** (0.03)	-0.25 ** (0.04)
HGT	-0.27 ** (0.19)	0.01 ^ns^ (0.22)	-0.09 * (0.21)	-0.20 ** (0.21)	-0.14 * (0.22)	-0.22 ** (0.22)	0.00 ^ns^ (0.04)	0.30 ** (0.04)	0.13 * (0.04)	0.02 ^ns^ (0.04)	-0.16 * (0.04)	0.06 ^ns^ (0.04)
VOL	-0.44 ** (0.19)	-0.17 ** (0.24)	-0.32 ** (0.22)	-0.41 ** (0.20)	-0.47 ** (0.19)	-0.49 ** (0.20)	-0.23 ** (0.04)	0.21 ** (0.04)	-0.07 ^ns^ (0.04)	-0.20 ** (0.04)	-0.45 ** (0.03)	-0.20 ** (0.04)

** statistically significant at α = 0.01

* at α = 0.05

^ns^ non-significant at α = 0.05

### Genetic gain and response to selection

Genetic gain and correlated genetic response to indirect selection for target traits using selection intensity of 1% is presented in Table 4. Selection based on VOL and DBH resulted in positive gain for all growth traits VOL, DBH and HGT as well as for MFA (ca 20%, 9%, 4% and 5%, respectively) and in negative gain for DEN and MOE_s_ (ca -3% and -6%, respectively). On the other hand, selection based on wood density traits (DEN, RES_TB_, PIL and PIL_B_) generated negative gain for growth traits and MFA and positive gain for wood quality traits. Selection for DEN led to its moderate genetic gain (9%) and a relatively big correlated response for MOEs (18%), MFA (-12%) and VOL (-13%), while selection based on RES_TB_, PIL and PIL_B_ had a lower impact on MOE_s_ (12%, 8% and 8%, respectively), MFA (-5%, -4% and -2%, respectively) and DEN (8%, 4% and 6%, respectively).

**Table 4:** Genetic gain (in parentheses) and correlated genetic response (both in %) for selected traits with selection intensity of 1%.

Selection traits	Target traits					
	VOL (20.04)	DBH (9.40)	HGT (7.52)	DEN (9.07)	MOE_s_ (19.27)	MFA (19.18)
DBH (9.40)	19.75	-	3.51	-2.90	-6.47	5.66
VOL (20.04)	-	9.29	4.32	-2.75	-6.26	5.35
DEN (9.07)	-12.94	-6.40	-2.34	-	17.56	-12.38
RES_TB_ (10.43)	-11.51	-6.01	-1.70	8.45	12.27	-4.71
PIL (9.21)	-11.41	-6.23	-1.01	4.45	7.71	-3.74
PIL_B_ (9.57)	-11.84	-5.74	-1.57	5.58	7.53	-1.61

## Discussion

### Predictability of wood density using Resistograph and Pilodyn

The relevance of inclusion of wood quality traits (such as density, stiffness or strength) into breeding programs of different conifer species, especially when genetic correlations between growth and wood quality traits are unfavorable, has been intensively studied in a number of species (e.g. [4, 8, 12, 15, 22, 28]), particularly in radiata pine [29–31]. Breeding strategies aiming at overcoming such adverse correlations have been designed [32, 33] and economic weights for optimal combination of growth and stiffness have also been applied in real breeding selection [34]. However, the time and cost demanding assessment of wood quality on standing trees, along with a low market pressure, often prevents wood quality traits from being taken into account during selection [15]. Nevertheless, in spite of the lack of interest from the current wood processing industry, breeders should anticipate prospective needs already now, as long-term breeding of forest trees is not capable of flexible responding to rapid technological advances and market changes [22]. Wood density is generally considered to be the most important wood quality trait because it greatly influences wood properties and performance and thus significantly affects wood suitability for different end uses. Integration of wood density into a breeding program, however, requires non-destructive screening of a large number of standing trees.

Pilodyn penetrometer was successfully applied for wood density estimation in some studies (e.g. [15, 35]) whereas it failed in other studies (e.g. [6, 36]). Application of Pilodyn is quick and simple but it has a shallow penetration, low sensitivity and gets affected by thick bark [19]. In this study, Pilodyn was applied for Scots pine wood density estimation of standing trees both with and without bark. Phenotypic correlations of inverse Pilodyn penetration (PIL) with SilviScan wood density (DEN) were weak, while the genetic correlation was moderate (*r*_*G*_ = 0.59) before and relatively strong (*r*_*G*_ = 0.74) after bark removal. Correlations of a similar magnitude were found in black spruce [18] or loblolly pine [37]. Nevertheless, Pilodyn does not seem to be a suitable tool for wood density assessment in Scots pine compared to e.g. Norway spruce or *Eucalyptus nitens*, for which strong genetic correlations were estimated (*r*_*G*_ = 0.96) [15, 35].

In contrast to Pilodyn, Resistograph micro-drill measures a whole stem profile with a high accuracy. Its main drawback is, however, a need for adjustment of drilling profiles that show an increasing trend as an effect of accumulated needle friction. Approaches for adjustment of Resistograph measurements vary among authors. For example, [38] and [39] applied centered moving averages and centered moving minima to smooth and detrend profiles, while [22] and [18] used only a few first centimeters of the profile and thus eliminated part of the profile exhibiting a steep increase.

In the present study, the whole Resistograph drilling profiles were detrended, assuming that their increasing trend was linear, and bark was excluded from both sides because bark can reach considerable thickness in Scots pine and thus can constitute a substantial portion of the drilling profile. A strong genetic correlation (0.89) between Resistograph and SilviScan densities was attained already with raw data, and an even stronger correlation (0.96) was reached after profiles’ adjustment. The effect of detrending & debarking was more pronounced at phenotypic correlations (0.59 → 0.72). The results indicate that an adjustment of raw drilling profiles is an important procedure to obtain more accurate wood density estimates and that the Resistograph is a reliable tool for non-destructive wood density assessment of standing Scots pine trees. Similar results were also reported e.g. for loblolly pine (*r*_*P*_ = 0.75, *r*_*G*_ = 0.92) [38] or maritime pine (*r*_*P*_ = 0.77–0.79, *r*_*G*_ = 0.96 – 0.98) [22].

### Variation in Resistograph density numbers

Resistograph measurements taken in the southeast direction (denoted as A) showed a higher variation and a lower minimum value compared with measurements taken from the southwest direction (denoted as B). This could be caused by wood distortion and/or wood deterioration in some of the assessed trees, as the ten-millimeter-cores extracted for SilviScan analysis were taken in the southeast direction four years earlier. The Resistograph was applied in a reasonably close proximity of healed boring holes with the intention to obtain profiles comparable to those from SilviScan but, at the same time, some distance (ca 5–10 cm) was kept to avoid drilling through wood affected by the increment core extraction.

### Heritability

Narrow-sense individual-tree heritability of Resistograph wood density varies between 0.14 and 0.64 [8, 18, 40]. In our study, heritability for adjusted Resistograph density was moderate (0.43), similar to that reported by [22] for maritime pine. Estimates of heritability for Pilodyn penetration range from very low to high. [41] reported individual narrow-sense heritabilities between 0.11 and 0.90 for Scots pine growing at different sites, while [37] between 0.06 and 0.46 for loblolly pine measured by different types of Pilodyn. A high heritability (> 0.6) was determined by [35] for *Eucalyptus nitens*. In this study, individual narrow-sense heritability of 0.32 was estimated for both Pilodyn penetration with bark and without bark. The results are similar to those reported for Norway spruce by [42] and [15].

### Correlations

We have determined negative genetic correlations between wood density and growth traits (-0.47 and -0.44 for DEN vs. DBH and VOL, respectively), which is well in line with earlier studies in Scots pine [3, 24, 43]. Genetic and phenotypic correlations of DBH and VOL with indirect wood density measurements (RES_TB_, PIL, PIL_B_) followed the same pattern as those with benchmark SilviScan. The only exceptions were phenotypic correlations of PIL, which were double compared to phenotypic correlations of PIL_B_ or DEN. We assume that trees with larger diameter have thicker bark and thus Pilodyn’s penetration is deeper as the pin has to penetrate through lower-density bark first before it reaches wood. That is the most plausible explanation of Pilodyn’s higher scores in larger trees, which, using the inverse of Pilodyn penetration depth, resulted in a stronger negative phenotypic correlation with DBH than for Pilodyn without bark (PIL_B_). Nevertheless, such a big difference between PIL and PIL_B_ did not emerge in genetic correlations. The effect of Resistograph profiles’ adjustment was clearly visible in the magnitude of genetic correlations and their standard errors. Genetic correlations of DBH and VOL with RES were weak, whereas they were moderate with RES_TB_ and very close to the benchmark values. A similar pattern between genetic correlations of growth traits with wood density estimated by Resistograph or Pilodyn and genetic correlations of growth traits with benchmark wood density were also published in other studies; either the correlations were moderately negative [12, 15, 18 for a mixed forest only] or near zero [38].

## Conclusion

In order to find a reliable tool for rapid and non-destructive wood density assessment of standing Scots pine trees, we tested penetrometer Pilodyn and micro-drill Resistograph using SilviScan wood density as a benchmark. As Pilodyn’s measurement is limited to a few centimeters of outer wood, we applied Pilodyn with and without bark, so that the influence of bark could be evaluated. According to our results, it seems that removing bark leads to more accurate estimates; nevertheless, the narrow-sense heritability remained unchanged. Phenotypic correlations of stem diameter with Pilodyn penetrations suggest that conducting the measurements with bark can result in underestimating of wood density as the lower-density bark might constitute a substantial portion of the measured profile. Unlike Pilodyn, Resistograph considers the whole stem profile and provides a detailed scan of wood density variation within it. Resistograms that exhibit increasing trend should be adjusted in order to improve accuracy of wood density estimates. We proposed linear detrending followed by bark removal as the accuracy of Resistograph wood density estimates substantially increased after the detrending and even slightly more after subsequent debarking. We also compared Resistograph’s measurements taken in two mutually perpendicular directions and concluded that a single measurement would be sufficient if a proper distance from knots or visible stem damages was kept. In summary, having compared Pilodyn and Resistograph wood density estimates with the benchmark wood density of SilviScan, it is obvious that Resistograph is a more suitable tool for wood density assessment in Scots pine standing trees (even before drilling profiles’ adjustment) than Pilodyn.
